# Attempt to Exterminate Mice by Means of the Shrew-mouse Bacillus

**Published:** 1898-01

**Authors:** S. S. Mereshkowsky

**Affiliations:** St. Petersburg


### ABSTRACTS FROM FOREIGN JOURNALS.

### GERMAN.

UNDER THE DIRECTION OF J. PRESTON HOSKINS, PH.D.,
PRINCETON, N. J.

ATTEMPT TO EXTERMINATE MICE BY MEANS OF THE SHREW-MOUSE BACILLUS. By S. S. Mereshkowsky, St. Petersburg.—Out of four kegs of barley-meal and two litres of liquid culture, Mereshkowsky made a dough, out of which 2000 little lumps were prepared. The culture of the shrew-mouse bacillus was developed for three days at a temperature of 35.5° C. These little lumps were devoured as greedily by the mice as wheat or corn.

During the harvest, while the grain was standing in shocks in the field, Mereshkowsky placed eight lumps in each shock. Two days later all these lumps had disappeared. Three days later the 250 shocks were opened, all the dead mice collected and all the live ones caught. A bacteriological examination gave the following result:

| | Total. | Infected. | Uninfected. | Per cent. of infected. |
|---|---|---|---|---|
| House-mice . . | 36 | 34 | 2 | 94 |
| Field-mice . . . | 36 | 22 | 14 | 61 |
| Wood-mice . . . | 15 | 12 | 3 | 80 |
| Total . . . . | 87 | 68 | 19 | 78 |

The percentage of dead animals was, in the case of field mice, 32 per cent.; house-mice, 29 per cent.; and wood-mice, 42 per cent.

II. On a second trial only four little lumps were put into sixty-two shocks, with the following result:

| | Total. | Infected. | Per cent. of infected. |
|---|---|---|---|
| Field-mice . . . . . | 85 | 36 | 42 |
| House-mice . . . . . | 14 | 6 | 43 |
| Wood-mice . . . . . | 12 | 9 | 75 |

Some seventy-five of these experiments were made, and the per cent. of infected mice steadily decreased, especially that of the house-mice, which sunk from 94 per cent. to 43 per cent. This fact was explained by the circumstance that the owner of the farm had ten dogs which received no regular feed and lived chiefly upon mice. This was confirmed by the fact that no dead mice were found on the outside of the shocks.

Mereshkowsky found that a second infection with the lumps should not be made until the end of twelve days, as it cannot be determined before that period whether all the infected mice have died. The bacillus has the same destructive effect upon rats, as was shown by examination, and the virus was often transmitted to the young, supposedly through the skin of the ovaries. Mereshkowsky comes to the conclusion that the bacillus taken from the shrew-mouse can be used for the extermination of the mouse-pest, yet the results will not be so rapid, because the number that can be infected is small compared with the whole number. It would, therefore, be advisable to infect all the places : fields, barns, cellars, etc., which are infested with mice.—*Deutsche Thierärzt Wochenschrift*, Jan. 30, 1897.

